# Adapting traditional content validation methods to fit purpose: an example with a novel video assessment and training materials in Duchenne muscular dystrophy (DMD)

**DOI:** 10.1007/s11136-019-02245-2

**Published:** 2019-07-13

**Authors:** Michelle K. White, Mindy Leffler, Kaitlin Rychlec, Chris Jones, Christine McSherry, Linsey Walker, Mark Kosinski

**Affiliations:** 1 Optum, Johnston, RI USA; 2 Casimir LLC, Bellevue, WA USA; 3 iTakeControl Health, Inc., Boston, MA USA; 4 Sarepta Therapeutics, Inc., Cambridge, MA USA

**Keywords:** DMD, Video, Technology, Assessment, Validation

## Abstract

**Purpose:**

The Duchenne muscular dystrophy (DMD) Video Assessment requires a caregiver to record six videos of their ambulatory child performing physical activities using a smartphone application (app). Innovative assessments that combine a performance measure, technology, and an observer require new approaches to content validation studies. This study presents a novel approach to establish the feasibility and content validity of the Video Assessment and usability of the app.

**Methods:**

Interviews used concept elicitation and an adapted cognitive debriefing approach. Interviews were conducted with 8 clinicians who reviewed training materials prior to the interview and provided feedback on the appropriateness and usefulness of the DMD Video Assessment and the relevance of the physical activities. Four caregivers reviewed training materials and used the app to record their child performing the physical activities prior to the interview. Researchers reviewed the recordings against a checklist to identify discrepancies in caregivers’ understanding of the training materials prior to an interview. During the interview, caregivers commented on comprehension of the materials, appropriateness of the activities, and feasibility of recording the videos.

**Results:**

Clinicians found the DMD Video Assessment and materials appropriate and useful for assessing disease progression and treatment response. Caregivers found the activities appropriate and the training materials and app easy to understand and use. Feedback resulted in changes to the training materials, but not the activities or videotaping procedure.

**Conclusions:**

Researchers used an innovative methodological approach that adapted traditional methods of content validation for the purpose of evaluating a technology-based performance measure in its totality. While future studies should be conducted with a larger, more diverse sample, these study findings add to our understanding of the content validity of the DMD Video Assessment.

## Introduction

Duchenne muscular dystrophy (DMD) is a rare, progressive, degenerative, and universally fatal neuromuscular disease with X-linked recessive inheritance. The disease is caused by mutations in the gene that encodes dystrophin, a large protein that stabilizes muscle fibers by linking intracellular actin filaments to the sarcolemma membrane and protects against mechanical damage during contraction [1, 2]. It affects approximately 1 in 3500 to 5000 males born annually worldwide [3–5]. The majority of boys with DMD (66–68%) have large deletions that involve at least 1 exon and disrupt the reading frame in the dystrophin mRNA, terminating translation of the dystrophin protein [1, 2, 6]. Loss of dystrophin compromises the regenerative ability of muscle fibers and leads to increased muscle membrane fragility, muscle necrosis, and fatty tissue replacement [6–8].

The progression of DMD follows a predictable course with irreversible decline [1]. Initially, boys gain in functional ability, albeit more slowly than unaffected peers, as they grow and develop during early childhood. However, as muscle deterioration overtakes muscle growth around age 7, they begin to experience a steady decline in function. This is marked by loss of ambulation and wheelchair dependence by early adolescence [1, 7].

Accurate and feasible measures of disease progression and treatment efficacy are needed for clinical practice and for measuring outcomes in research studies. Current efficacy measures used in clinical trials, such as the 6-minute walk test [9], the North Star Ambulatory Assessment [10], the Performance of Upper Limb test [11], and the Brooke Upper Extremity Scale [12] do not capture subtle changes in function. Clinical evaluations are comprehensive but also lengthy, costly, inconvenient, and thus infrequently conducted [13].

To date, patient- and observer-reported outcome measures have not been adequately integrated as standardized clinical assessments of DMD treatments, as only a small number of published studies have reported findings based on these types of outcome measures in DMD [9, 14–16]. Innovative outcome measures are needed to broaden the understanding of the impact of DMD on daily life as well as to ascertain how well new treatments may preserve daily functioning. Such measures need to be easily administered and convenient so as to facilitate the accumulation of more robust data on the natural history of the disease. This will provide a better account of the degree of heterogeneity in functioning observed across individuals.

The DMD Video Assessment is a novel measurement strategy developed in 2014 by advocates in the DMD community as a measure of physical function of ambulatory boys participating in clinical trials. The Video Assessment approach has a parent (or another caregiver) record and submit videos of their child performing six typical physical activities using a smartphone application (app). In response to recommendations from Food and Drug Administration (FDA), Casimir LLC (Casimir) developed a written instruction manual and training videos that describe procedures to ensure standardization and consistency across recordings.^1^

**Fig. 1:**
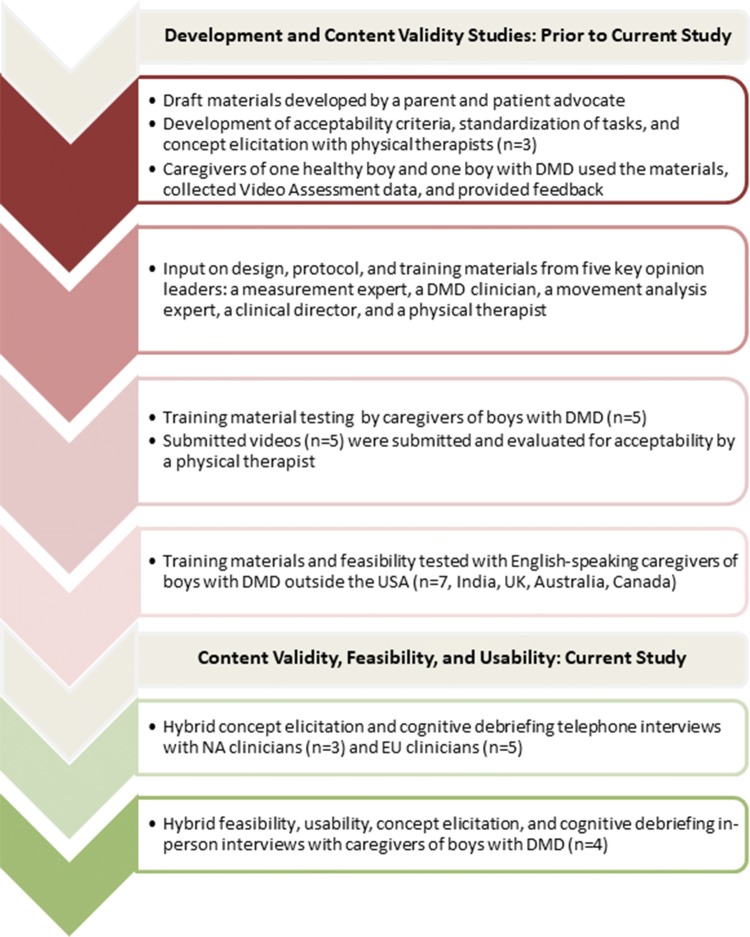
DMD Video Assessment development, content validity, feasibility, and usability

Establishing content validity is a critical step in outcome measure development. Content validity is most reliably established with evidence that the content measures concepts that are appropriate and understood relative to the intended measurement concept, population, and use [17–19]. The DMD Video Assessment developers conducted a series of initial concept elicitation and pilot testing studies that resulted in revisions of draft materials (Fig. 1). In all, the revisions incorporated input from physical therapists and clinicians, movement experts, caregivers of boys with DMD, and a measurement expert.^2^ Additional pilot tests of the Video Assessment and materials with caregivers within the US and internationally were conducted and reviewed for acceptability by physical therapists. The resulting set of materials provided strong evidence of content validity for use with boys with DMD, but the developers recognized a need to fill gaps in the evidence. Gaps included the small number of clinicians involved in testing, and that the pilot testing with caregivers was informal. Caregivers received the training materials and other supplies with no additional instruction, collected data using the Video Assessment, submitted the videos, and then provided unstructured feedback to the developers on the experience.

Performance-based outcome assessments, especially those that incorporate technology, present a unique challenge. Evaluation of content validity not only requires the traditional steps necessary for evaluation, but also requires innovative approaches to confirm appropriateness, comprehensibility, and feasibility of the assessment and the accompanying materials. This manuscript provides an example of how our research team adapted typical methods of evaluating content validity to fill the gaps in evidence of the DMD Video Assessment.

## Methods

### Participants

Eight clinicians (3 North American [NA] and 5 European [EU]) and four caregivers were recruited. Clinicians were eligible to participate if they were currently treating boys with DMD, had previously treated at least 15 DMD patients, and had participated in at least one DMD-related clinical trial. Attempts were made to recruit clinicians from a range of backgrounds, including diversity of geography, gender, and years in practice. Caregivers were recruited through outreach by a patient advocacy group, the Jett Foundation, which used their contact list to identify potential caregivers in the Boston area. Caregivers were eligible to participate if they were able to read and speak English, had access to an Apple or Android phone and home internet service, and were a primary caregiver of an ambulatory boy with DMD who was diagnosed more than 1 year prior, 7 years of age or older, and able to follow caregiver directions such as to walk up stairs or stand up.

### DMD Video Assessment

The DMD Video Assessment is a performance-based measure that requires a parent or other caregiver to record six short videos of the child performing the following typical daily physical activities: walking (front view), walking (side view), sitting up, standing up, ascending and descending stairs, and an activity of choice (selected by the caregiver). The selected activity of choice should remain the same during repeated assessments. These activities were selected by the Video Assessment developers based on their experiences with their children, who have DMD, as being typical daily activities that would be negatively affected by DMD disease progression and could be positively affected by a treatment. The activities are common to clinicians treating DMD, can be performed in the home, and can be easily videotaped using a smartphone app. The Video Assessment includes activities that are appropriate for the ambulatory population.^3^

The DMD Video Assessment is accompanied by a written caregiver instruction manual and a set of training videos. The manual describes how to prepare for recording (equipment needed, lighting, timing, location, minimization of distractions, and clothing) and contains specific instructions for each activity (including how to ensure the correct elements of movement are captured). The training videos provide similar information in video format and show examples of a boy with DMD performing the activities. Caregivers use a secure, authenticated smartphone app to record and upload the videos.

### Study procedures

Two sets of interviews were conducted consecutively to evaluate the content validity of the DMD Video Assessment: (1) interviews with clinicians in NA and the EU; and (2) interviews with caregivers in the USA.

The clinician interviews were conducted by phone in March and May 2017. Approximately 7 days prior to the interview, clinicians received the written manual, training videos, and instructions describing their pre-interview review activities. This approach ensured clinicians had adequate time to consider the materials and had viewed videos taken using the training materials to ensure they fully understood the measure. The interviewer obtained consent from each clinician to audio record the interview.

The interviewer used a semi-structured interview guide that began with open-ended questions followed by systematic questions on each aspect of the written manual, training videos, and each activity. The clinicians were asked to offer comments on the relevance of each activity and were encouraged to provide suggested changes where appropriate. Next, they were asked questions intended to gauge the feasibility and usefulness of the DMD Video Assessment in drug development clinical trials and in clinical practice. Specific questions were added to assess relevance and comprehension specific to different regions. Subsequent interviews incorporated feedback from earlier interviews.

The caregiver interviews were conducted in June 2017. Prior to receiving any study materials, all participants were emailed an informed consent form and provided verbal consent.^4^ Approximately 10 days prior to the scheduled interview, participants were emailed the instruction manual, instructions for downloading the Video Assessment app, and a pre-interview instruction sheet outlining the following activities to be completed in their home at least 3 days prior to the interview:

- Download the app
- Read the Video Assessment instruction manual
- Watch the training videos in the app
- Record and submit the six videos in the app at least 3 days prior to interview

The day before the interview, the interviewer reviewed the submitted videos and compared them to the standard demonstration training videos using a checklist of criteria of required elements for each videotaped activity. The acceptability criteria checklist was designed to evaluate how well participating caregivers videotaped each of the physical activities according to the training materials (Table 1). During the pre-interview review, the interviewer took notes on discrepancies in the recording of the activities or production of videos submitted and during each interview probed about any areas of concern. This step was critical for discovering ways in which caregivers deviated from the training protocol or had difficulty understanding instructions, but hadn’t realized or reported it during the interview.

**Table 1:** DMD Video Assessment acceptability criteria

I. General rating criteria
• Clarity of video focus and lighting
• Patient’s whole body visible in frame
• Dress code followed: no orthoses, aids, or footwear (including socks)
• Proper verbal instruction given
• Reason for skipped videos recorded (if applicable)

II. Specific video criteria: patient compliance with assessment instructions
• Walking: front view
Adequate length of walking path
Child walks to and from camera
• Walking: side view
Adequate length of walking path
Child walks on walking path through frame *only*
• Sitting up
Child starts lying down on floor with arms at sides
Activity filmed until child reaches sitting position
• Standing up
Child starts sitting cross-legged, with arms crossed
Activity filmed until child reaches standing position
• Stairs
Adequate number of stairs recorded (at least 5)
Child filmed walking up and down stairs
• Choice activity
Appropriate activity chosen
Activity filmed from appropriate angle, with whole body visible in frame

Each interview began with open-ended questions to establish rapport and continued using a semi-structured interview guide to gather specific information regarding comprehension of the written manual, training videos, and to elicit feedback on their experience recording the videos. This design allowed for probing and exploration in instances where clarification was needed or unexpected themes emerged. Later interviews incorporated feedback from earlier interviews. This allowed for confirmation of suggestions from as many caregivers as possible. Each interview was audio-recorded with the consent of the caregiver.

### Data coding and analysis

All interview recordings were transcribed verbatim. Transcripts were coded by each element of the DMD Video Assessment: general feedback, instruction manual, training videos, and each activity video using Excel or NVivo (QSR International Pty Ltd., Melbourne, VIC, Australia, Version 10, 2012) databases. Clinician interview data coding and interpretation was completed prior to development of materials for the caregiver interviews.

The data coding team included:

1. study PI, a trained qualitative researcher and measurement expert with over 20 years of experience, who conducted all of the interviews
2. primary coder, a trained outcomes research associate with several years of experience
3. secondary coder, a trained outcomes research associate with several years of experience
4. scientific advisor, a trained researcher and measurement expert with over 30 years of experience
5. developer of the Video Assessments measure

The following description of coding procedure refers to the numbers in the list above. First, transcripts were reviewed for accuracy by coding team member #1 (study PI). Team member #2 coded all transcripts. Team member #3 reviewed the coding files by checking them against transcripts and entered any discrepancies into an Excel file for discussion. The study PI (#1) then reviewed all coding files and the discrepancy list. Easily resolved discrepancies were addressed; then the full coding team (members #1–5) met to discuss and resolve the few remaining discrepancies. The developer of the DMD Video Assessment (#5) also reviewed the coding summary file. Once coding was complete, feedback was analyzed by the study PI (#1) and primary coder (#2) to identify emerging and confirmatory themes, areas of confusion or disagreement, and recommendations for changes. All feedback was carefully considered. Analysis and recommendations were reviewed by the full team until a list of findings and recommended changes had been finalized for each set of interviews.

## Results

### Participant characteristics

Clinician sample characteristics are provided in Table 2. Caregiver sample characteristics are provided in Table 3.

**Table 2:** Clinician sample characteristics

Demographic information, *N* = 8	*N* (%)
Years in practice (post-residency)	
5 to less than 10	1 (12.5)
10 to less than 20	2 (25)
20 to less than 30	4 (50)
30 or more	1 (12.5)
Number of patients with DMD treated	
51 or more	8 (100)
Number of DMD clinical trials	
2 to 4	2 (25)
5 or more	6 (75)
Geographic region of primary practice	
Canada	1 (12.5)
France	1 (12.5)
Germany	1 (12.5)
Israel	1 (12.5)
Italy	1 (12.5)
United Kingdom	1 (12.5)
United States	2 (25)
Gender	
Female	4 (50)
Male	4 (50)

**Table 3:** Caregiver sample characteristics

Demographic information, *N* = 4	*N* (%)
Caregiver characteristics	
Relationship to child with DMD	
Mother	4 (100)
Highest level of education	
Bachelor’s degree	3 (75)
Post-graduate degree	1 (25)
Patient characteristics	
Age of child with DMD (years)	
9	2 (50)
11	1 (25)
12	1 (25)
Approximate time since diagnosis (years)	
6	1 (25)
7	2 (50)
8	1 (25)

### Relevance of physical activities selected for DMD Video Assessment

All clinicians found the DMD Video Assessment relevant and agreed the specific physical activities are appropriate for ascertaining disease progression and have a high correlation with many major milestones of DMD. Clinicians especially liked the use of the smartphone app and the ability for caregivers to record the videos in the convenience of their home; however, one clinician expressed concern that some families may have limited access to the necessary electronic devices.

Caregivers said the videos would be useful to improve communication with clinicians. All caregivers indicated having no problems motivating their child to participate, but thought that other children with DMD could become frustrated or refuse to participate.

### Feedback on the caregiver instruction manual and training videos

All clinicians found the instructions clear, detailed, and easy to understand. They recommended providing metric equivalents for video instructions involving distances for non-USA instructions, adding distances to the figures displayed in the instructions, adding more pictures to the instruction manual for additional clarity, and simplification of verbal instructions given to the child with DMD prior to recording the physical activities. Clinicians thought the training videos would be useful in helping caregivers correctly record their child’s physical activities in a standardized way. Clinicians reported that watching the videos and reading the training manual prior to the interviews was helpful and engaging.

Caregivers found the instruction manual and the training videos useful and easy to understand. All caregivers preferred using both the written instruction manual and the training videos. While all caregivers understood the instructions for skipping and re-recording videos, one caregiver suggested an option that says, “My child can no longer perform this” and another caregiver suggested adding a statement to the instructions to allow enough time to record each activity. Caregivers reported that actually using the training materials and app and recording the videos provided better insight for the interviews.

### Feedback specific to each physical activity

Clinicians and caregivers confirmed the importance and relevance of the six activities. Below are considerations for each activity.

#### Walking (front view)

Two clinicians noted the standard walking distance used in clinics is 10 m (about 33 feet); one recommended changing the distance to 10 m to allow for better comparison. Another clinician was concerned that some families may not have 25 feet available to use as a walking path, but that a shorter path might be adequate. Similarly, three caregivers reported problems with finding a walking path of 25 feet. Two were able to find a path in their homes and one filmed the walking videos outside and noted this would not be feasible in poor weather.

#### Walking (side view)

Comments from clinicians and caregivers on walking (side view) mirrored those offered for front view. Most clinicians were surprised by the inclusion of side view walking specifically, but endorsed it as important. One clinician noted that toe-walking and lordosis are assessed better from the side. One clinician and one caregiver recommended adding an instruction to hold the phone in landscape view while videotaping this activity.

#### Sitting up

Two caregivers suggested the need for additional clarity in the instruction for this activity: one was not sure if her son was allowed to use a chair to help him sit up, and the other put a towel on the floor for her child’s comfort but wasn’t sure if that was allowed.

#### Standing up

Clinicians noted that standing up is often measured in clinical trials. Several clinicians noted that standing up with arms crossed would be very difficult, and the instructions to try to keep them crossed may frustrate many children. One clinician thought this video should be filmed from farther away than is specified in the instructions. Caregivers recognized the potential difficulty that this task may pose for their children and suggested that specific instruction be added to use a chair or other object to help them stand up if needed.

#### Stairs

One clinician recommended that children should be instructed to “try not to use the rail.” Two clinicians reported that four steps are used in clinical trials, but that consistency for the same child across administrations is the most important in terms of the number and height or length of steps. One clinician suggested having caregivers use a ruler to indicate the height of a step to help ameliorate the issue of different caregivers having access to different step heights. The availability of stairs in the home was a common concern for all the EU clinicians. They also raised concerns with variability in stair height.

The stairs activity elicited the most concern, and even anxiety, from caregivers. They were particularly worried about how much longer their son would be able to go up and down stairs. One caregiver recorded this video at the physical therapist’s office because she did not have stairs in her house. Two caregivers were not sure if the use of the hand rails was allowed. One of the two opted to tell her son to “do whatever you need to do.” The other suggested adding, “If your child has to sit down to come down the stairs or crawl to go up the stairs, that’s okay.” A third caregiver told her son it was okay to crawl, because he had completely lost the ability to walk up the stairs. One caregiver thought five steps was “extreme,” and suggested that three steps might be better.

#### Choice activity

All of the clinicians liked the idea of the choice video. Several clinicians suggested providing more examples in the instructions. Caregivers described feeling anxious when selecting an activity. Caregivers selected examples provided in the instruction manual for the choice activity or asked the child pick the activity rather than thinking of something specific for their child. Several caregivers recommended the written manual provide additional examples.

### Videos to add

Clinicians and caregivers were asked if they would recommend adding activities to the Video Assessment. While many activities were suggested, there was no consensus. Clinicians expressed concerns over caregiver and patient burden if activities were added. Caregivers felt the choice video could be used instead of adding another activity.

### Feasibility and comprehension of training materials

Overall, each of the videos submitted by the caregivers met the criteria (see Table 1) used to evaluate the quality of the video recordings. All four caregivers successfully submitted the videos using the app. The mean time required to record and submit all six videos was 35 min (range 15–60 min, excluding one caregiver who had technical difficulties uploading the videos). It should be noted that time to complete was expected to be a bit longer than would be observed with continued use. Overall, caregivers complied with recording directions. Only one caregiver deviated from the instructions: during the recording of the walking videos, the caregiver only had the patient walk in one direction and not the other (instructions state to walk in both directions). Caregivers noted their smartphones timed out or locked prematurely, which was related to their phones default settings rather than the app, suggesting the written manual or app instruct the caregiver to adjust those settings prior to videotaping.

## Discussion

The DMD Video Assessment is a novel, performance-based measure that allows caregivers to record subtle changes in their child’s physical activities using a smartphone app. Repeated assessment allows for identification of changes in quality of movement, including using more or fewer compensatory movements to complete an activity. The instruction manual and training videos within the app should ensure that the videos are captured in a standardized and consistent way. The review of training materials by clinicians confirmed the validity, feasibility, and usefulness of the Video Assessment in clinical practice.

An innovative methodological approach to cognitive debriefing interviews with caregivers was required due to the nature of the assessment and technology. Caregivers were asked to review training materials and use the app to record their child carrying out the six activities prior to the interview. The interviewer reviewed and evaluated submitted videos using predetermined acceptability criteria checklist to inform the interview and verify caregiver feedback. This method allowed caregivers to more fully participate in cognitive debriefing interviews, provided the interviewer with a deeper knowledge of the participant experience, and allowed for easy rapport building. It also allowed the interviewer to use probes to remind caregivers of any deviations they had not themselves noticed. In this study there were few serious deviations not spontaneously reported. Studies with participants who have less technological ability or with children with co-occurring DMD and attention-deficit/hyperactivity disorder (ADHD) or other commonly co-occurring behavioral conditions may result in a higher number of identified discrepancies. It should be noted that the training materials in this study were pilot tested several times prior to the study with feedback already implemented; this likely impacted the number of discrepancies identified.

Overall, the results of this qualitative evaluation did not find any critical need to change the specific physical activities of the DMD Video Assessment nor the videotaping procedures. Most suggestions were made by only one or two study participants, but all suggestions were considered by the research team due to the small number of participants. Clinicians and caregivers agreed the specific physical activities captured were important and relevant for measuring disease progression. There was clear recognition that the purpose of the DMD Video Assessment is not to assess whether a child is able to perform the physical activity, but instead is to assess the *quality* of movement during the activity, including compensatory movements used during the activity. Clinicians thought this was vital to measuring subtle changes in disease progression.

Interviews with caregivers confirmed recording these six activities was feasible and they had little difficulty using the app to record and transmit the video recordings. Three of the four caregivers correctly interpreted and implemented the instructions for all six videos, and the fourth had errors in two of the six videos. Caregivers reported reading the training manual more than once and having some questions about what to do. The video instructions were very helpful, but in some cases limited what caregivers thought was acceptable for their child in terms of modifications or compensatory movements their child made to perform certain activities.

Several clinician and caregiver recommendations should be considered to improve the training materials. First, the instruction for both walking videos requires a 25-foot clear path within the home, which was of concern to both clinicians and caregivers. Their suggestion was to shorten the length of the walking distance since, as one clinician stated, “It is not how far they can walk, it is whether the image is clear in the video and the number of steps that the child takes is adequate for the evaluators to get a sense of how the child is walking.” Also, concerns were raised by clinicians and caregivers regarding the climbing stairs activity because many families do not have stairs in their home. This detracts from the convenience of the DMD Video Assessment as a measure that can be collected at home and potentially introduces other concerns such as having to videotape this activity on a different day than the other physical activities, or videotaping in a public area. Videotaping in public may make it impossible to have a clear view without other people in the recording and introduces privacy concerns. It is not known how the performance of one activity affects the child’s performance of other activities that are part of the DMD Video Assessment. Therefore, if the timing of each videotaped activity relative to the other activities is variable, the ratings across children may be confounded. Another concern raised with the climbing stairs activity was the variability in the height of steps accessible to these families using the Video Assessment. Such differences could confound comparisons of performance ratings across children on this physical activity. While this activity was deemed important to ascertain the degree of disease progression, clinicians recommended caregivers have the option to skip this video or offer an alternative physical activity in its place. Last, caregivers preferred that the Video Assessment instruction manual provide additional examples of activities for the choice activity, as they struggled to think of an activity.

One of the most important features of the DMD Video Assessment is the convenience to caregivers to be able to use their own smartphones in their homes. An often-expressed hardship on the family is the travel expense and time required to go to the clinic for assessment. Consequently, clinic visits happen less frequently, often once or twice per year. The DMD Video Assessment allows clinicians to assess performance of physical activity more frequently, which facilitates the capture of data that reflect more subtle changes in performance over time and contributes to a better understanding of the natural history of the disease as it progresses.

This study had a small number of caregiver participants; however, confirmation that caregivers could easily interpret the instruction manual and correctly record the videos was an important piece of evidence of the content validity of the DMD Video Assessment. Any ambiguity in the interpretation of instructions or inability to follow the instructions could impact the quality of the video recordings, and thus the validity of the measure. In addition, the concept elicitation portion of the interviews further confirmed the concepts measured in the six activities as being appropriate for ambulatory boys with DMD.

While this study engaged multiple clinicians from North America and several countries in Europe, only four caregivers, all of whom lived on the east coast in the USA, participated, limiting study findings. These caregivers may have had access to more resources or may in other ways not be representative of all caregivers of boys with DMD. For example, all caregivers were mothers. Future studies should involve other caregivers. In addition, inclusion criteria for the caregivers specified having the ability to clearly read and speak English. Future studies should include caregivers for whom English is a second language and those who have difficulty reading. Since the training materials also include a video that shows a child performing the activities, this may be less of a concern. Finally, these particular caregivers had the ability to travel to the interview, had good internet service, and a sufficient smartphone to download and use the app. Future studies should incorporate caregivers who may not have the same resources. Clinical sites or research studies that wish to use the DMD Video Assessment may need to provide a smartphone, Internet access, initial training, or other support.

Despite these study limitations, results from this qualitative study provide an example of how researchers adapted typical cognitive debriefing methods to consider the uniqueness of this performance-based Video Assessment that requires use of training materials, technology (smartphone app), and a caregiver as data collector. The pre-interview procedures and acceptability criteria checklist were important for evaluating not just what the caregivers remembered having trouble with or thinking they might have trouble with, but what they actually did have trouble understanding or implementing. The findings from this study, when added to the findings from prior rounds of evaluation and testing, provide evidence of the appropriateness of the concepts measured, the feasibility of procedures for recording, the usability of the smartphone app, and the comprehensiveness and comprehensibility of the training materials. The DMD Video Assessment is a novel approach to use technology to better identify changes in disease progression among ambulatory boys with DMD.

## Footnotes

1 Leffler M., McSherry C. (2017). Caregiver assessments for the ESSENCE clinical trial program: report on Casimir validation efforts. [Summary available from author upon request.].

2 See footnote 1.

3 Because the disease trajectory of DMD includes loss of ambulation, a non-ambulatory assessment is under development.

4 Caregivers provided written consent at interview location.
